# *Candida*–Epithelial Interactions

**DOI:** 10.3390/jof4010022

**Published:** 2018-02-08

**Authors:** Jonathan P. Richardson, Jemima Ho, Julian R. Naglik

**Affiliations:** Mucosal & Salivary Biology Division, Dental Institute, King’s College London, London SE1 1UL, UK; jemima.ho@kcl.ac.uk (J.H.); julian.naglik@kcl.ac.uk (J.R.N.)

**Keywords:** epithelial cell, *Candida*, fungus, mucosal infection, commensalism, pathogenicity, microbiota

## Abstract

A plethora of intricate and dynamic molecular interactions occur between microbes and the epithelial cells that form the mucosal surfaces of the human body. Fungi, particularly species of *Candida*, are commensal members of our microbiota, continuously interacting with epithelial cells. Transient and localised perturbations to the mucosal environment can facilitate the overgrowth of fungi, causing infection. This minireview will examine the direct and indirect mechanisms by which *Candida* species and epithelial cells interact with each other, and explore the factors involved in the central processes of adhesion, invasion, and destruction of host mucosal surfaces.

## 1. Introduction

The mucosal surfaces of the human body are colonised by a rich and diverse microbiota, placing them under relentless microbial scrutiny. Fungi are an important but often overlooked component of the microbiota [1,2,3] that colonise mucosal surfaces, and one of the most common of these species are the *Candida* spp. There are over 150 spp. of *Candida* fungi, of which approximately 20 are known to be pathogenic to humans. While *Candida* spp. are typically commensal organisms, several factors, including alterations in the host immune status, microbial dysbiosis, lifestyle choices (e.g., smoking), and genetic predisposition can facilitate fungal overgrowth and a switch to pathogenic behaviour. Although *Candida albicans* is regarded as the most common of the *Candida* spp, *C. glabrata*, *C. tropicalis*, *C. parapsilosis*, and others are increasingly isolated from mucosal surfaces [4]. Indeed, the prevalence of *Candida* spp. and their continually evolving threat to human health is underpinned by the recent emergence of *C. auris* [5].

In order to colonise a host, *Candida* spp. must first interact with a mucosal surface. *Candida* spp. use physical attributes and an array of proadhesive, proinvasive and damage-inducing factors to facilitate colonisation and infection, and in this minireview, we will discuss the role of these factors in *Candida*–epithelial interactions.

## 2. Adhesion of *Candida* Species to Epithelial Surfaces

Microbial adhesion to epithelial surfaces is an essential prerequisite for mucosal colonisation and infection. The contact with the epithelium can be mediated through direct physical attachment or indirectly through physical association with co-colonising microbes and/or abiotic substrates. The site of fungal attachment can serve as a staging post for superficial, deep-seated, and systemic infections with high mortality in susceptible individuals. The adhesion of *Candida* spp. to epithelial cells is a complex, dynamic, and multifactorial process defined by the intimate association between components of the fungal cell wall and epithelial surface proteins.

Initial interactions between *C. albicans* yeast and host epithelium occur through the passive processes of hydrophobic and electrostatic attraction [6]. Changes in cell surface hydrophobicity correlate with changes in external protein content [7] and the ability of *C. albicans* to adhere to epithelial cells [6,8]. Moreover, hydrophobic yeasts are more virulent than hydrophilic yeasts in mice [9].

Once in contact with the epithelium, some *Candida* spp. undergo morphological switching from yeast to hyphae, and this morphological transition is a major virulence trait of some, but not all, *Candida* spp. For example, while *C. albicans*, *C. dubliniensis*, and *C. tropicalis* form hyphae, which adhere more strongly to epithelial cells than yeast cells [10], other species such as *C. glabrata* and *C. parapsilosis* do not form hyphae but are still adherent.

*C. albicans* Hyphal Wall Protein 1 (Hwp1p) is a key hypha-associated adhesin that facilitates epithelial cell attachment and is highly expressed during colonisation and infection of the oral mucosa [11,12]. The N-terminus of Hwp1p is a substrate for mammalian transglutaminase enzymes, resulting in covalent cross-linking of *C. albicans* to the oral epithelium [13]. These interactions preferentially occur within the outermost, terminally differentiated layers of the stratified oral epithelium that display the differentiation markers keratin 13 and small proline-rich protein 3 (SPR3) [14]. Deletion of *HWP1* results in reduced adhesion to oral epithelial cells and decreased virulence in a murine model of oropharyngeal candidiasis (OPC) [15]. In addition to Hwp1p, *C. albicans* Hwp2p has also been reported to be required for adhesion to HT-29 human epithelial cells in vitro [16].

The *C. albicans* Als (agglutinin-like sequence) proteins [17], comprise an eight-member family of cell surface adhesins (Als1–7p and Als9p) which are GPI-linked to β-1-6 glucans in the fungal cell wall. The Als protein family has conserved amyloid-forming regions, and Als1p, Als3p, and Als5p contain the same heptapeptide amyloid-forming sequence [18]. Als-dependent cellular adhesion is concomitant with increases in cell surface hydrophobicity [19], suggesting that the amyloid-forming regions of Als proteins may contribute to overall hydrophobicity. Indeed, it is now understood that the formation of amyloids is an intrinsic property of yeast cell adhesin proteins [20], and the activation of discrete amyloid nanodomains within adhesins is required for cellular attachment [21]. *C. albicans* Als3p (hypha-associated) is a major epithelial adhesin that is strongly upregulated during epithelial infection in vitro [12], and disruption of the *ALS3* gene reduces epithelial adhesion in vitro. Likewise, decreasing the expression of the *ALS2* gene also reduces adhesion [22,23]. Interestingly, deletion of the *ALS5*, *ALS6*, or *ALS7* genes was observed to increase adhesion [24], indicating that the Als proteins can have opposing roles in mediating fungal attachment to mucosal surfaces. However, the role of certain Als proteins during epithelial interaction remains controversial, with conflicting reports regarding the contribution of Als1p, Als2p, and Als4–6p [22,25,26].

Putative homologues of Als proteins have also been identified in *C. parapsilosis*, *C. dubliniensis*, and *C. tropicalis* although there is significant sequence divergence between spp. [27,28] and limited experimental data regarding their role in epithelial adhesion. However, the deletion of a *C. parapsilosis* homologue of *C. albicans* Als3p (*CPAR2_404800*) resulted in reduced adhesion to buccal epithelial cells in vitro and diminished pathogenicity in a murine model of urinary candidiasis [29], suggesting that at least some of these homologues have similar roles.

While *C. albicans* and *C. dubliniensis* differ considerably in terms of their pathogenicity, their genomes are remarkably similar [30]. The similarity between genome sequences is broadly conserved across the *ALS* gene family with the exception of *ALS3*, which is absent from *C. dubliniensis* [30]. Indeed, it has been suggested that divergent evolutionary trajectories (gene deletions including *ALS3* for *C. dubliniensis* and expansion of a telomere-associated (TLO) gene family of transcriptional regulators within *C. albicans*) may account for the differences in pathogenicity between species [30,31].

The role of *C. albicans* Hyphally Regulated protein (Hyr1p) in epithelial adhesion is less clear, although a role in biofilm formation has been established. Disruption of the *HYR1* gene leads to reduced virulence in vivo [32]. Other proteins not associated with hypha formation are also involved in *C. albicans* adhesion. For example, Eap1p (Enhanced Adherence to Polystyrene) is required for biofilm formation and adhesion to epithelial and fungal cells [33,34,35], while the GPI-linked, cell wall-associated aspartic proteinases Sap9p and Sap10p have opposing roles in epithelial adhesion: deletion of the *SAP9* gene from *C. albicans* increases adhesion, while deletion of the *SAP10* gene reduces adhesion [36]. Overexpression of the GPI-anchored IPF Family F protein Iff4p increases adhesion of *C. albicans* to epithelial but not endothelial cells [37].

In contrast to *C. albicans*, the main adhesion proteins mediating attachment of the non-filamentous fungus *C. glabrata* are Epithelial Adhesin (Epa) and Epa-like proteins. Epa1p of *C. glabrata* is a 1034-amino acid GPI-anchored, glucan cross-linked, cell wall-associated lectin required for robust, calcium-dependent adhesion to laryngeal and hamster ovary epithelial cells in vitro [38]. Epa1p binds to asialo-lactosyl-containing carbohydrates on host cells. An *EPA1* gene deletion mutant exhibited a 95% reduction in epithelial adhesion, identifying this factor as a major contributor to *C. glabrata* adherence. Indeed, Epa1p alone is sufficient to mediate adhesion to epithelial cells, as expression of *EPA1* in *Saccharomyces cerevisiae* resulted in robust epithelial adhesion [38].

Intriguingly, Epa1p-mediated adhesion to epithelial cells appears to be strain-dependent [39]. Some strains of *C. glabrata* rely on Epa1p for the majority of their epithelial adhesion, while others have minimal dependence. *C. glabrata* contains a number of *EPA1* gene paralogues located at the subtelomeric regions of its genome [40], and different strains of *C. glabrata* express differing repertoires of Epa adhesins [41], some of which (Epa6p and Epa7p) are regulated by subtelomeric silencing [42]. Given the large size of the *EPA* gene family in *C. glabrata* (>20 *EPA*-like members), it is likely that a certain degree of functional redundancy may account for the differences in experimental observations. Indeed, a degree of molecular promiscuity is apparent in the adhesin domain of Epa1p, Epa6p, and Epa7p that facilitates attachment to a number of different carbohydrate ligands, particularly those that contain terminal galactose residues [43,44]. Common adhesins used by *Candida* spp. to interact with epithelial cells are presented in Table 1.

## 3. Induced Endocytosis

Following colonisation, *Candida* spp. can invade the epithelium in order to establish an infection. *C. albicans* can enter epithelial cells by a passive host-mediated process called induced endocytosis [45,46]. Endocytosis of *C. albicans* hyphae and non-filamentous *C. glabrata* occurs within 4 h of initial contact with epithelial cells [47,48]. Induced endocytosis is triggered by the recognition of invasins expressed on the fungal cell surface. To date, two *C. albicans* invasins have been identified, Als3p and Ssa1p, both of which interact with the host epithelial receptor E-cadherin [49,50]. Interaction of E-cadherin with Als3p or Ssa1p promotes the accumulation and colocalisation of dynamin, clathrin, and cortactin at the site of hyphal contact, which coordinate the remodelling of the actin cytoskeleton required to endocytose the fungus [51]. *C. albicans als3∆/∆* and *ssa1∆/∆* gene deletion mutants exhibit reduced binding to, and invasion of epithelial cells and are attenuated for virulence in a murine model of OPC [49,50,52].

Induced endocytosis can also occur independently of epithelial E-cadherin through the interaction of fungal Als3p and Ssa1p with the epidermal growth factor receptor (EGFR/HER2) heterodimer expressed on epithelial cells [53] and through a mechanism involving host GTPases (Cdc42, Rac1, RhoA) and the tight junction protein ZO-1 [54]. The PDGF BB (platelet-derived growth factor BB) and NEDD9 (neural precursor cell-expressed developmentally downregulated protein 9) pathways are also implicated in epithelial uptake of *C. albicans*, as siRNA knockdown of PDGF receptor beta (PDGFRB) or NEDD9 causes a reduction in endocytosis [55]. More recently, the host epithelial aryl hydrocarbon receptor (AhR) has been identified as an important upstream component involved in fungal endocytosis [56]. Activation of AhR by *C. albicans* results in Src-mediated phosphorylation of EGFR and fungal internalisation, while inhibition of AhR was observed to reduce invasion and OPC severity [56].

Intriguingly, some *Candida* spp. exploit the process of induced endocytosis to avoid immune recognition. For instance, *C. parapsilosis* is efficiently endocytosed by endothelial cells which provide a means of escape from patrolling neutrophils [57]. However, it must be noted that this interaction occurs within the systemic compartment and its relevance to mucosal responses has yet to be established.

## 4. Active Penetration

The oral and vaginal mucosae are comprised of stratified layers, the outermost of which are terminally differentiated, nonproliferative, and therefore less likely to support fungal invasion through induced endocytosis. *Candida* spp. must therefore use an alternative mechanism to invade a tissue that does not readily support internalisation. This process is called active penetration. Active penetration of mucosal barriers requires a viable fungus and occurs by hyphal invasion through or between epithelial cells. Active penetration is dependent upon fungal attributes including hyphal turgor pressure, physical advancement of the hyphal tip, and secretion of factors such as hydrolytic enzymes that may facilitate the breaching of mucosal barriers by degrading host substrates. The *C. albicans*-secreted aspartic proteinase Sap5p can degrade E-cadherin, an important component of epithelial adherens junctions [58], while Sap2p can degrade mucins which coat all mucosal tissues [59]. Active penetration is mechanistically distinct from induced endocytosis, as blocking actin polymerisation does not prevent mucosal penetration from occurring [52,60]. While proteinases are thought to play a significant role in *C. albicans* invasion and penetration, secreted lipases and phospholipases appear to play a limited role in these processes [60,61]. *C. parapsilosis* has been observed to invade the connective tissue of human oral mucosa [62], although the mechanism of invasion has yet to be characterised. Clinical isolates of *C. parapsilosis* and *C. tropicalis* also colonise and invade reconstituted human oral epithelium [63,64] and both display strain-dependent variation in their ability to do so.

Active penetration of *C. albicans* hyphae occurs at all mucosal surfaces, but in the gut it is the sole driver of invasion across the epithelium [49,60]. Active penetration occurs at later time points during infection when compared with induced endocytosis. Although induced endocytosis is observed to occur prior to active penetration in vitro, active penetration is likely to be the initial mechanism that enables *C. albicans* to invade through the outermost layers of an epithelium in vivo. However, once the fungus has accessed the underlying proliferative layers of an epithelium, induced endocytosis may then occur to further facilitate invasion. While induced endocytosis and active penetration are therefore mechanistically distinct processes, both are nevertheless likely to be required to establish infection through stratified mucosal barriers in vivo.

## 5. Epithelial Interactions with *Candida* Species

The process of receptor-mediated epithelial recognition of *Candida* spp. is poorly understood, but recent studies have shed light on the signalling pathways activated by *Candida*, particularly *C. albicans*. *C. albicans* yeast cells weakly activate three key signalling pathways: all three MAPK (mitogen-activated protein kinase) pathways (namely, p38, JNK, ERK1/2), the NF-κB (nuclear factor kappa-light-chain-enhancer of activated B cells) pathway, and the PI3K (phosphatidylinositide 3-kinase) pathway [65,66]. This drives the activation of the transcription factors NF-κB and c-Jun (via ERK1/2 and JNK) but is insufficient to induce immune activation. *C. albicans* hyphae activate the same signalling pathways, but there is a far stronger activation of MAPK signalling, with specific induction of the transcription factor c-Fos (via p38) [65]. *C. albicans* hyphae also activate MKP1 (MAPK phosphatase 1) via the ERK1/2 pathway, which is known to regulate MAPK-mediated immune responses [65]. This combination of c-Fos and MKP1 activation is specifically associated with hypha formation and correlates with immune activation. While the PI3K pathway is also involved in immune activation, its main function appears to be in protecting epithelial cells against hypha-induced damage [66].

The epithelial receptors that interact with *C. albicans* to initiate these signalling mechanisms and induce immune responses are unclear. Epithelial cells express numerous pattern recognition receptors (PRRs) (e.g., toll-like receptors (TLRs), C-type lectin receptors), including TLR2, TLR4, and dectin-1 [62,65,67,68], which can alter in expression after *Candida* challenge [62,66]. These PRRs recognise yeast and hyphal cells via conventional fungal pathogen-associated molecular patterns (PAMPs) (i.e., mannans, β-glucans) [69,70]. However, this conventional PAMP–PRR interaction does not appear to activate the c-Fos/MKP1 signalling pathway or the secretion of immune modulatory cytokines [65,67]. Notably, both c-Fos/MKP1 signalling and immune activation have recently been shown to be activated by a novel peptide toxin called Candidalysin that is secreted by *C. albicans* hyphae [71] (see below). However, recently it has been suggested that the β-glucan of *C. glabrata*, *Rhizopus delemar*, *Aspergillus fumigatus*, *S. cerevisiae*, and *C. albicans* yeast and hyphae can interact with the epithelial PRR Ephrin Type-A Receptor 2 (EphA2), activating signal transducer and activator of transcription 3 (STAT3) and MAPK signalling that culminates in proinflammatory antifungal responses [72]. This indicates that dectin-1 may not be the only PRR that recognises β-glucan and further suggests that epithelial cell recognition of fungal PAMPs may be different to myeloid cell recognition of fungal PAMPs. As such, while it is clear that dectin-1 plays a crucial role in systemic *Candida* infections [73,74,75,76], its role in mucosal infections and immune responses appears minimal [65]. This was elegantly demonstrated recently by Gaffen and colleagues who showed that protective innate Type 17 responses against oral candidiasis were independent of dectin-1 but dependent upon Candidalysin activity [77].

## 6. Secreted Factors, Nutrient Acquisition, and Damage

Numerous factors secreted by *Candida* spp. are capable of interacting with epithelial cells, including hydrolytic enzymes and toxins. Sap-like enzymes are present in several *Candida* spp., including *C. dubliniensis*, *C. tropicalis*, and *C. parapsilosis* [30,78,79,80,81]. The loss of these secreted factors can significantly impact on the interactions between these fungi and epithelial cells. For example, deletion of *C. albicans SAP1*, *SAP2*, or *SAP3* genes has been observed to reduce adherence to buccal epithelial cells, while a *sap4-6*Δ/Δ triple mutant displayed increased adhesion [82]. Collectively, the SAP family exhibits broad substrate specificity [83] and can degrade epithelial cadherin [58] and gastrointestinal mucins [59], which may yield metabolically useful substrates during the course of an infection.

The secreted phospholipases of *C. albicans* have been implicated in adhesion to buccal epithelial cells, although their role is somewhat controversial [61,84,85]. Strain-dependent phospholipase and proteinase activity has also been detected in the emerging pathogen *C. auris* [86]. Deletion of the *C. parapsilosis* lipases *CpLIP1* and *CpLIP2* inhibits biofilm formation, reduces virulence on reconstituted human oral epithelium, and increases susceptibility to killing by immune cells [87], demonstrating a role for these enzymes in mucosal pathogenicity.

During mucosal infection, the hyphae of *C. albicans* secrete Candidalysin, a cytolytic peptide toxin derived from the hypha-associated protein Ece1p. The Candidalysin toxin is a dual-function molecule. As mentioned above, Candidalysin can induce mucosal immunity predominantly through the activation of MAPK signalling via c-Fos and MKP-1. In addition, Candidalysin also damages the plasma membrane of epithelial cells [71]. It is tempting to speculate that Candidalysin may facilitate fungal acquisition of nutrients by damaging epithelial membranes, causing the release of substrates that are metabolically useful to the invading fungus. 

In this regard, it is interesting to note that Als3p expressed on *C. albicans* hyphae can sequester ferritin from epithelial cells [88]. The *C. albicans* CFEM (Common in Fungal Extracellular Membranes) proteins Rbt5p, Pga7p, and Csa1p also play a role in iron acquisition from host proteins [89,90]. Compared with *C. albicans*, the CFEM family is expanded in *C. parapsilosis* [91]. The *CFEM2* and *CFEM3* genes of *C. parapsilosis* are critical for haem utilisation, whereas the *CFEM6* gene is only partially required [91].

**Figure 1:**
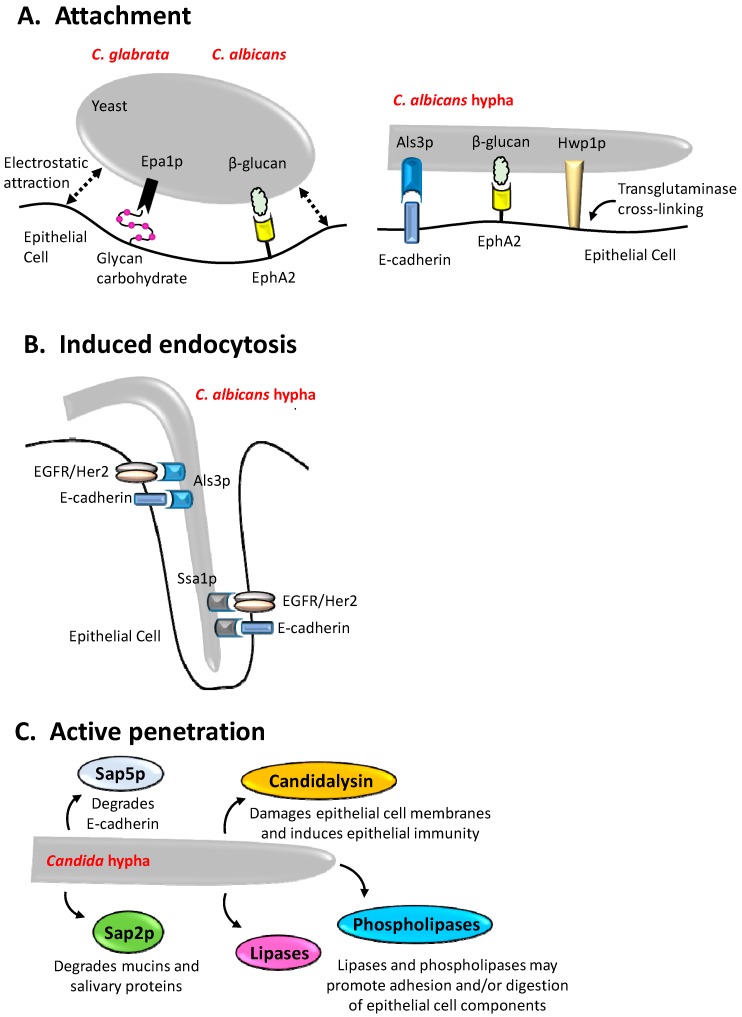
Summary of *Candida* and epithelial cell factors directly involved in attachment, entry, and damage to epithelial cells. (**A**) The prototypical *Candida* species *C. albicans* can attach to epithelial cells through numerous host cell receptors including EphA2 (via β-glucan) and E-cadherin (via Als3p). Host cell transglutaminases also cross-link *C. albicans* directly to the epithelial surface (via Hwp1p), whilst the non-filamentous *C. glabrata* can utilise Epa1p to bind host-cell glycans (asialo-lactosyl-containing carbohydrates). Additionally, electrostatic forces (dashed lines) contribute to the overall affinity between fungal and host cells; (**B**) *C. albicans* Als3p and Ssa1p interact with E-cadherin and EGFR/Her2 receptors to potentiate induced endocytosis; (**C**) several *Candida* species such as *C. albicans*, *C. dubliniensis*, and *C. tropicalis* secrete factors to actively penetrate mucosal tissues, including aspartic proteinases (Sap2p, Sap5p), lipases, phospholipases, and Candidalysin, predominantly from hyphae.

The ability to acquire and assimilate host micronutrients including copper and zinc has a profound influence on fungal pathogenicity [92]. Indeed, the importance of micronutrients is such that host cells and tissues have evolved mechanisms to modulate the availability of these essential nutrients in a process collectively referred to as nutritional immunity [93]. Secreted factors such as *C. albicans* pH-regulated antigen 1 (Pra1p) is required to scavenge zinc from endothelial cells [94], while zinc limitation results in altered cellular morphology in several *Candida* spp. (*C. albicans*, *C. dubliniensis*, and *C. tropicalis*, but not *C. parapsilosis* or *C. lusitaniae*) and increased adhesion of *C. albicans* to abiotic substrates [95]. Factors that contribute to epithelial interactions are presented in Figure 1.

## 7. Heterotypic Interactions between *Candida* Species and Mucosal Bacteria

It is becoming increasingly clear that the microbial communities that colonise mucosal surfaces have a profound influence on *Candida*–epithelial interactions. Adhesion of *Candida* spp. to a mucosal surface can be direct (via classical adhesins for example) but can also occur indirectly through specific interactions with other fungi and bacteria [96,97].

The analysis of mixed fungal cultures using a magnetic bead-based adherence and aggregation assay revealed that *C. albicans* can form heterotypic aggregates with *C. glabrata* and *C. parapsilosis* in vitro [98]. Infection of tongue tissue by *C. glabrata* during murine OPC is enhanced when co-infected with *C. albicans* or when added to a pre-established *C. albicans* infection [99], strongly suggesting that an initial epithelial interaction with one spp. of *Candida* can have a dramatic influence on the pathogenicity of a different fungal spp. Such interactions are not limited to other fungi, however. *Streptococcus gordonii* is a primary commensal coloniser of the oral cavity, and *C. albicans* Als3p can interact with the SspB adhesin of *S. gordonii* [100], while Als5p is also capable of binding to *S. gordonii* [98]. Als3p also mediates binding between *C. albicans* and *Staphylococcus aureus* [101].

The interaction of *Candida* spp. with one another and with mucosal bacteria may serve to increase the likelihood of a successful *Candida*–epithelial interaction, although this is not always the case. For instance, the probiotic bacterium *Lactobacillus rhamnosus* GG induces a metabolic reprogramming of *C. albicans* causing reduced hyphal extension, adhesion to, and invasion of oral epithelial cells in vitro [102], demonstrating that the interactions between *C. albicans* and mucosal bacteria can have variable outcomes in the context of mucosal interaction.

## 8. Conclusions

*Candida* species employ a diverse range of direct and indirect mechanisms that enable the interaction with host epithelial cells. Under suitably predisposing conditions, commensal colonisation of mucosal surfaces is followed by pathogenic infiltration and secretion of hydrolytic enzymes and toxins that reduce mucosal barrier function, facilitating disease progression.

Importantly, variations between different *Candida* spp. and between fungal strains of the same species have an undoubted impact on *Candida*–epithelial interactions. For instance, while *C. albicans* triggers more epithelial cell damage than other *Candida* spp., the extent of damage varies between strains [103], leading to differences in alarmin production [83,104]. Likewise, differences in epithelial damage, invasion, and secretion of proinflammatory cytokines is also apparent between strains of *C. glabrata* [105], while different strains of *C. albicans* exhibit variable pathogenicity in animal models [103,106,107].

The physical contact between *Candida* spp. and members of the resident microbiota can influence the mucosal interactions and has direct consequences on disease outcome. In response, the epithelial receptor-mediated recognition of *Candida* spp. activates a number of dynamic host signalling pathways that enable appropriate proinflammatory and immune defence mechanisms to be implemented. The environmental conditions greatly affect the outcome of any experiment, including *Candida*–host interactions. The precise factors that influence these interactions are unquestionably variable and complex.

Numerous aspects of *Candida*–epithelial interactions remain to be explored in full. The majority of experimental data obtained thus far describes factors utilised by *C. albicans*, but there is an increasing need to identify the proteins used by other *Candida* spp. that influence epithelial interactions. It is likely that there are many more epithelial-specific receptors involved in *Candida* recognition that have yet to be identified and characterised. The signalling responses induced by fungal endocytosis have yet to be characterised in full, and the contribution of secreted factors to fungal pathogenicity remains an area of intense study. Indeed, while Candidalysin has been shown to contribute to both oral and vaginal infections in mice [71,108], the role of Candidalysin in the systemic compartment is not yet clear.

While considerable progress has increased our understanding of the complex relationship that exists between *Candida* fungi and mucosal surfaces, continued research will undoubtedly provide further clarity into the mechanisms that underpin this important host–pathogen interaction.

## Figures and Tables

**Table 1:** *Candida* species adhesins and their role in epithelial attachment.

Species	Gene	Function	Epithelial Adhesion of Null Mutant	Epithelial Cell Type	Reference
***C. albicans***	*ALS1*	Adhesin	Decreased	Tongue	[25]
	*ALS2*	Adhesin	Decreased *	Reconstituted human oral epithelium	[23]
	*ALS3*	Adhesin (hypha-associated)	Decreased	Buccal	[22]
	*ALS5-7*	Adhesin	Increased	Buccal	[24]
	*EAP1*	Adhesin	Decreased	HEK293	[34]
	*HWP1*	Cell wall protein (hypha-associated)	Decreased	Buccal	[13]
	*HWP2*	Cell wall protein	Decreased	HT-29	[16]
	*SAP9*	Aspartic proteinase	Increased	Buccal	[36]
	*SAP10*	Aspartic proteinase	Decreased	Buccal	[36]
***C. glabrata***	*EPA1*	Adhesin	Decreased	Laryngeal, Hamster ovary	[38]
	*EPA6*	Adhesin	Overexpression in *S. cerevisiae* confers adhesion	Lec2	[42]
	*EPA7*	Adhesin	Overexpression in *S. cerevisiae* confers adhesion	Lec2	[42]
***C. parapsilosis***	*CPAR2_404800*	Adhesin	Decreased	Buccal	[29]

* Heterozygous knockout only (*als2*Δ/*ALS2*).
